# Ultrasound-based attenuation imaging for assessing steatosis severity in overweight/obese children: a prospective single-center study

**DOI:** 10.1186/s13244-026-02291-8

**Published:** 2026-04-27

**Authors:** Tingting Liu, Murong Chen, Longlong Huang, Yanbing Lin, Shasha Huang, Meixi Chen, Zhe Su, Luyao Zhou, Wenying Zhou

**Affiliations:** 1 Department of Ultrasound, Shenzhen Children’ Hospital, No. 7019, Yitian Road, Futian District, Shenzhen, P.R. China; 2 Department of Endocrinology, Shenzhen Children’ Hospital, Shenzhen, P.R. China

**Keywords:** Child, Obesity, Overweight, Metabolic-associated steatotic liver disease, Attenuation imaging

## Abstract

**Objectives:**

To prospectively evaluate the correlation between the attenuation imaging (ATI) parameter and hepatic steatosis in overweight (OW)/obese (OB) children, and to establish normal ATI reference values from a prospectively enrolled cohort of healthy children.

**Materials and methods:**

A total of 653 prospectively enrolled children were categorized into OW, OB, and normal control groups based on body mass index (BMI). Ultrasonographic hepatic steatosis grading and ATI measurements were independently assessed by two radiologists. Hepatic steatosis was graded visually as none, mild, moderate, or severe.

**Results:**

The final study cohort consisted of 97 OW, 292 OB, and 264 control children. Median attenuation coefficient obtained with ATI for normal control group, OW group, and OB group were 0.51, 0.54, and 0.64 dB/cm/MHz, respectively. Statistically significant differences in ATI values were observed among all three groups (all *p* < 0.001). In the combined OW/OB subgroup, ATI values demonstrated a significant weak to strong positive correlation with age, height, weight, BMI, skin-to-liver distance, serum alanine aminotransferase, aspartate aminotransferase, triglycerides, and uric acid (all *p* < 0.05). Additionally, ATI values increased stepwise with the severity of hepatic steatosis and showed a statistically significant positive correlation with steatosis grade, with higher grades corresponding to greater ATI values (η² = 0.626, *p* < 0.001).

**Conclusions:**

ATI values exhibit a significant stepwise increase across healthy, OW, and OB pediatric cohorts, and correlate with anthropometric/metabolic profiles and ultrasonographic steatosis severity. This evidence positions ATI as a non-invasive tool to grade severity and monitor treatment response in metabolic-associated steatotic liver disease.

**Critical relevance statement:**

ATI shows significant increases across pediatric weight groups, correlating with metabolic profiles and steatosis severity, positioning it as a non-invasive metabolic-associated steatotic liver disease assessment tool.

**Key Points:**

- The ATI value increased significantly in a stepwise manner from healthy controls to OW and OB children, confirming its sensitivity to fat-related liver changes.
- ATI correlates significantly with most metabolic and anthropometric parameters in OW and OB children, suggesting its utility in reflecting metabolic status.
- ATI values increase progressively with hepatic steatosis severity and show a strong positive correlation with ultrasonographic steatosis grade.

**Graphical Abstract:**

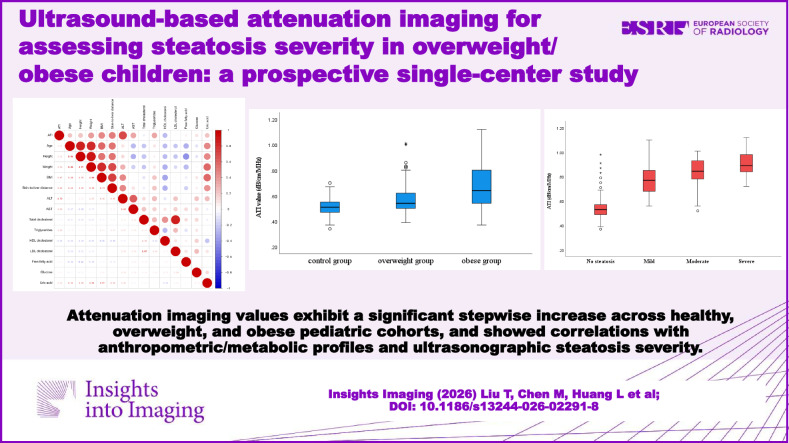

## Introduction

Childhood obesity has become an increasingly prevalent issue worldwide, leading to a rising incidence of metabolic-associated steatotic liver disease (MASLD) [1, 2]. Obesity is a major risk factor for MASLD, which affects 50% to 80% of overweight (OW) and obese (OB) children and has emerged as a leading cause of chronic liver disease in this population [3]. MASLD is associated with hepatic steatosis and can progress to metabolic-associated steatohepatitis and even cirrhosis, resulting in significantly higher mortality rates among affected children compared to their healthy peers [4, 5]. Early detection and timely intervention are crucial to prevent severe outcomes. Moreover, there is an urgent need for diagnostic tools capable of quantitatively assessing hepatic steatosis to monitor changes in liver fat during treatment.

Liver biopsy and magnetic resonance imaging of liver proton density fat fraction (MRI-PDFF) are considered the gold standard for the diagnosis and evaluation of the severity of MASLD [5, 6]. However, in clinical practice, liver biopsy is not recommended as a routine diagnostic tool for children suspected of MASLD due to its invasive nature and sampling error [6]. MRI-PDFF is also not recommended as a screening tool to detect MASLD in OB children because it is expensive [2, 5]. Ultrasound (US) is known as the first-line tool to screen liver diseases, including MASLD, in children due to its non-radiation and cost-effectiveness [7]. However, the US is not able to precisely quantify the grades of hepatic steatosis and is consequently unable to quantitatively monitor the changes in liver fat during the treatment process in children with MASLD [8]. It is crucial to develop a non-invasive strategy capable of quantitatively assessing the severity of hepatic steatosis.

Attenuation imaging (ATI) is a quantitative ultrasonic technology based on quantifying the attenuation of sound waves in the liver tissue, calculating the attenuation coefficient (AC) of the liver by calculating the echo signal, so as to reflect the acoustic attenuation changes caused by steatosis [9, 10]. As a quantitative complement to conventional US, ATI combines the practical advantages of US—such as portability, low cost, and wide availability—with objective and reproducible fat quantification, positioning it as a promising screening tool for early identification of children at high risk of MASLD. At present, ATI has been validated for the diagnosis of hepatic steatosis in adults, and relevant studies have shown that ATI can detect and quantify hepatic steatosis and distinguish different steatosis grades in adults [9, 11–14]. However, only a few studies have investigated the utility of ATI for the evaluation of fatty liver in the pediatric population [15, 16]. ATI values in healthy children and in children with hepatic steatosis are not well established yet.

This study aims to prospectively evaluate the correlation between ATI and hepatic steatosis in OW and OB children, and to establish normal ATI reference values from a prospectively enrolled cohort of healthy children.

## Methods

### Study design and patients

This single-center cross-sectional study was approved by the Institutional Review Board of Shenzhen Children’s Hospital (approval no. 2022131). In accordance with ethical guidelines, written informed consent was obtained from the legal guardians of all participants (under 18 years of age) prior to enrollment.

**Fig. 1:**
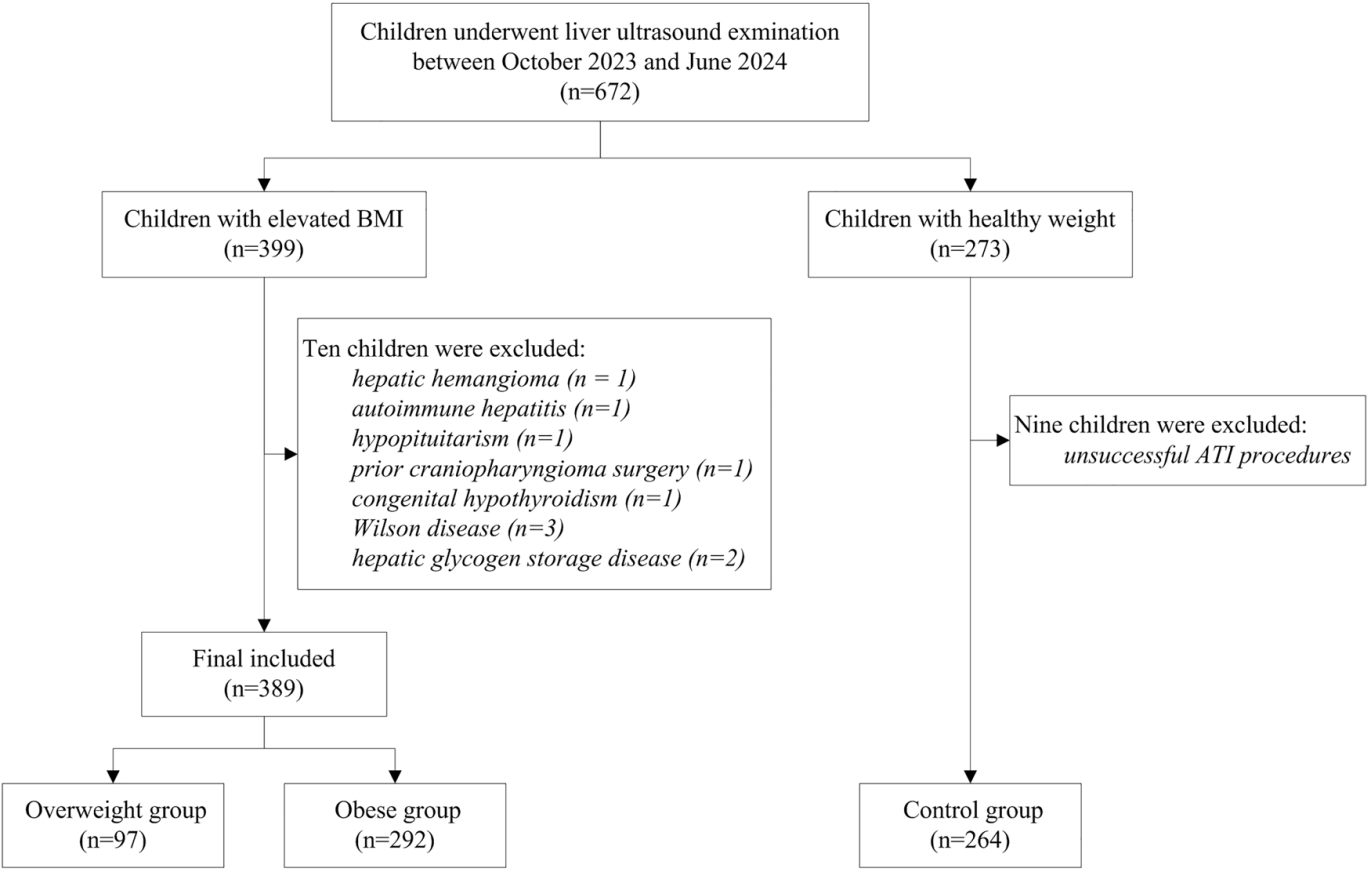
Patient flow diagram

A total of 399 children with elevated body mass index (BMI) were prospectively enrolled between October 2023 and June 2024 (Fig. 1). This cohort was stratified into OW and OB groups based on established BMI criteria, defined in accordance with the 2018 Health Industry Standard of the People’s Republic of China (WS/T 586-2018: Screening for OW and Obesity in School-Age Children and Adolescents) [17]. Participants in the OW/OB groups were required to undergo liver US examination with ATI assessment. Exclusion criteria included: (a) history of liver disease, (b) systemic conditions affecting hepatic function, (c) focal liver lesions, or (d) unsuccessful ATI acquisition. Based on the presence or absence of hepatic steatosis detected by US, the OW/OB participants were further classified into two subgroups: a non-steatosis group and a steatosis group.

To establish normal reference values for ATI in the pediatric population, a control group consisting of 273 children with normal weight was also enrolled. Eligibility for the healthy control group required: (a) age- and sex-appropriate BMI within the normal range, (b) serum liver function tests and lipid profiles within reference limits, and (c) normal liver US findings (including ATI). No steatosis was observed in any of the children in the control group.

### US assessment

**Fig. 2:**
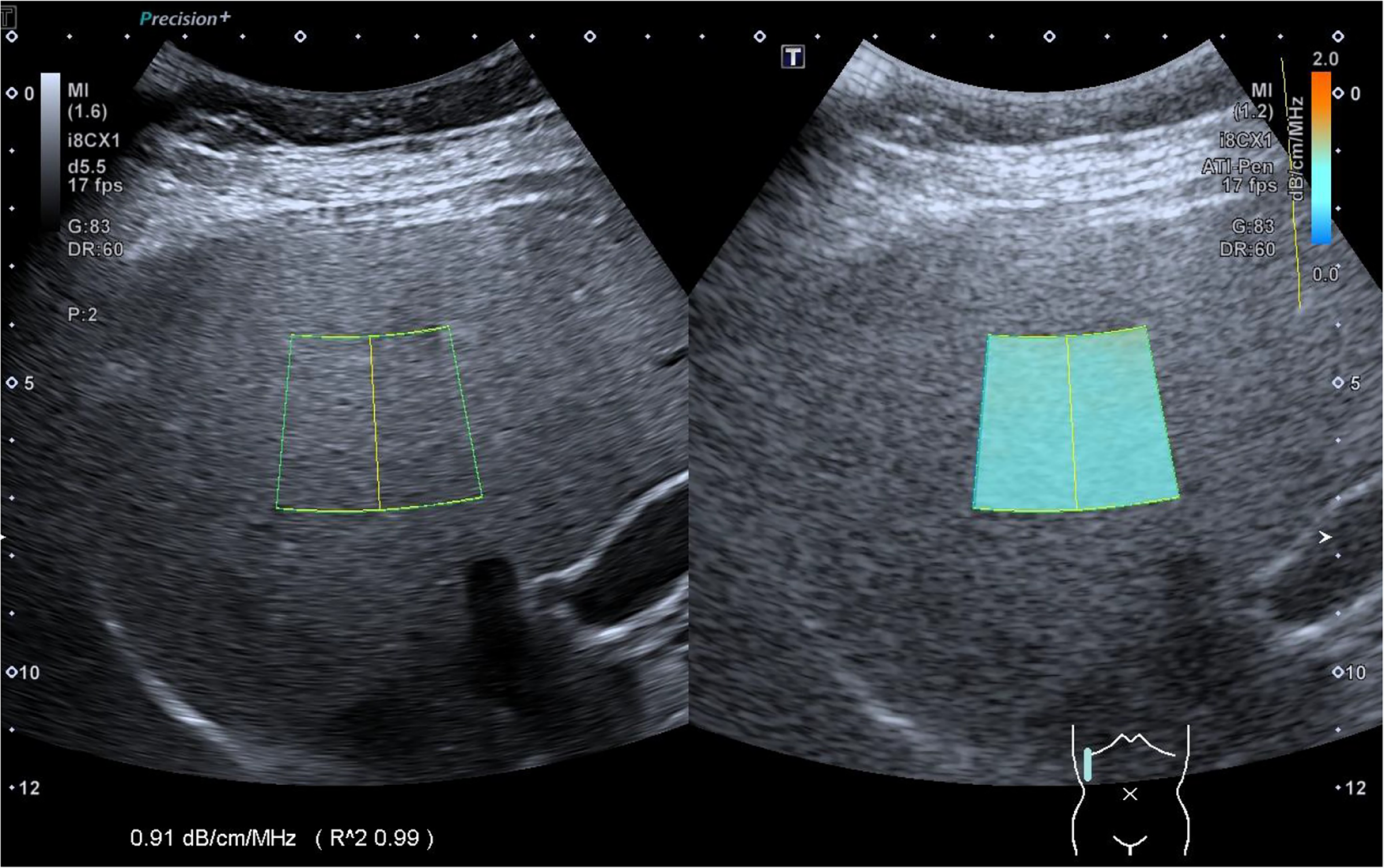
Performance of US ATI. A representative sonographic ATI image from a 10-year-old boy with hepatic steatosis, showing an ATI value of 0.91 dB/cm/MHz. The associated *R*² value of 0.99 indicates excellent reliability of the measurement

All US examinations were conducted using a Canon Aplio i900 US system equipped with an i8CX1 transducer. Two experienced radiologists (T.L. and M.C., each with > 8 years of pediatric US expertise) performed the scans. Radiologists were blinded to the patient’s clinical details. All patients needed to fast for at least 4 h before the US examination. The standardized ATI scanning protocol involved positioning the patient supine with their right arm elevated above the head, utilizing a right intercostal approach [18]. The transducer was maintained perpendicular to the liver capsule with an imaging depth of 12 cm. A fan-shaped sampling box (2.5 × 4.5 cm or larger) was positioned 1.0–1.5 cm from the liver capsule to minimize reverberation artifacts. The region of interest (ROI; at least 2.0 × 3.0 cm) was placed in an area free from signal interference, such as reverberation artifacts and acoustic shadowing. All measurements were acquired during a neutral breath-hold. The measurement result was shown as the ATI value (dB/cm/MHz) (Fig. 2).

The reliability of the measurements was assessed using the *R*² value, which was classified as poor (*R*² < 0.80), good (0.80 ≤ *R*² < 0.90), or excellent (*R*² ≥ 0.90). In accordance with the manufacturer’s specifications, measurements with *an R*² value of 0.80 or greater were considered valid. The ATI protocol required acquiring five valid measurements, and the median of these five values was used for subsequent analysis. The distance from the skin to the liver capsule was also recorded for each measurement.

When technically feasible and with sufficient patient cooperation, shear wave elastography (SWE), shear wave speed (SWS), and shear wave dispersion (SWD) were performed alongside the conventional US and ATI measurements. Patients with valid data from these assessments constituted a predefined subgroup for analyzing associations with ATI.

### The visual grade of hepatic steatosis

**Fig. 3:**
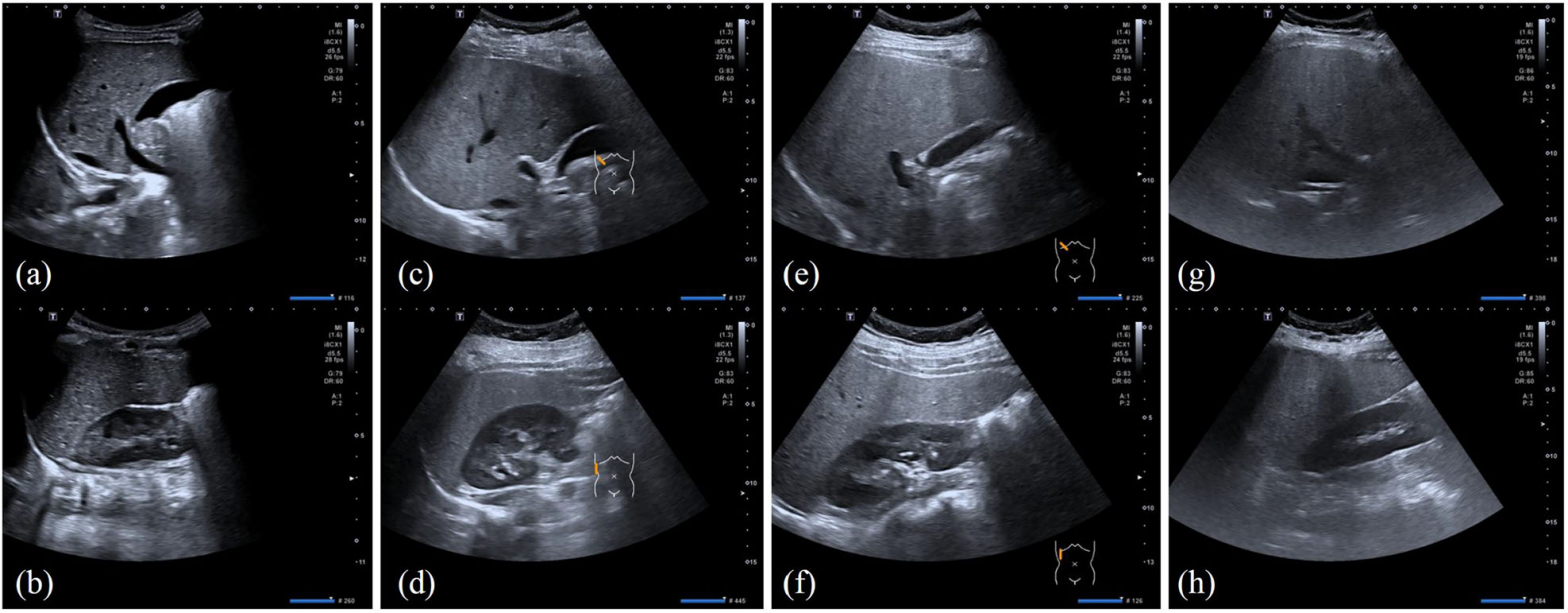
US images of normal (**a**, **b**), mild (**c**, **d**), moderate (**e**, **f**), and severe (**g**, **h**) hepatic steatosis

Hepatic steatosis was graded by radiologists based on standardized US features including liver echogenicity, liver-to-kidney echogenicity ratio, vascular border clarity, and diaphragm visualization [8, 19, 20] (Fig. 3). The grading system was defined as follows: Grade 1 (mild) steatosis was characterized by diffusely enhanced hepatic echogenicity exceeding that of spleen and kidney while maintaining clear visualization of intrahepatic ducts and diaphragmatic borders. Grade 2 (moderate) cases demonstrated hepatomegaly with near-field echo enhancement, mild far-field attenuation, and slightly impaired visibility of intrahepatic structures and diaphragm, though these remained discernible. The most severe Grade 3 presentation showed marked hepatomegaly with significant near-field echo enhancement, substantial far-field attenuation, and obscuration of both intrahepatic ductal structures and diaphragmatic margins.

The visual grading of hepatic steatosis severity was independently assessed by two other radiologists (L.H. and L.Z., each with over 10 years of pediatric US experience). All radiologists were board-certified in pediatric radiology with specialized expertise in pediatric liver US. When there was disagreement between the two diagnostic results, the decision was made after consultation. To ensure objective assessment, these two senior radiologists were blinded to other clinical data during image interpretation.

## Statistical analysis

The normality of continuous variables was assessed using the Kolmogorov–Smirnov test. Data with a normal distribution were expressed as mean ± standard deviation and compared using the Student’s *t*-test or one-way ANOVA. In contrast, non-normally distributed data were presented as median (interquartile range) and compared using the Mann–Whitney *U*-test or the Kruskal–Wallis *H*-test. Categorical variables were summarized as frequencies and percentages and analyzed by the chi-square test. The correlation between quantitative continuous variables and categorical variables was examined using Eta-squared (η²). The Spearman’s rank correlation coefficient was applied to assess the relationship between two non-normally distributed continuous variables. All statistical tests were two-sided, and a *p*-value of less than 0.05 was defined as statistically significant. All analyses were performed using SPSS software package version 27 (IBM).

## Results

### Patient characteristics

Among the 399 children with elevated BMI initially enrolled, 389 (97.5%) met the inclusion criteria and were included in the final analysis (Fig. 1). The final cohort comprised 97 children classified as OW and 292 as OB. Ten children were excluded for the following reasons: hepatic hemangioma (*n* = 1), autoimmune hepatitis (*n* = 1), hypopituitarism (*n* = 1), prior craniopharyngioma surgery (*n* = 1), congenital hypothyroidism (*n* = 1), Wilson disease (*n* = 3), and hepatic glycogen storage disease (*n* = 2). Additionally, after excluding 9 children due to unsuccessful ATI procedures, 264 children with a healthy weight were included as the healthy control group. The 9 healthy-weight children who were excluded presented with notably thinner abdominal walls and poorer cooperation, which resulted in inadequate acoustic windows during ATI examination and prevented the acquisition of stable, interpretable images. Baseline characteristics of all participants are summarized in Table 1.

**Table 1:** Characteristics of participants in the OW, OB, and control groups

characteristics	OW group *n* = 97	OB group *n* = 292	Control group *n* = 264	*p*-value
Male/female	79/18	221/71	153/111	0.016
Age (year)	11.5 (10.2, 13.1)	10.9 (9.1, 12.6)	10.5 (8.3, 12.8)	< 0.001
Height (cm)	152.7 (143.3, 164.9)	150.0 (140.0, 163.0)	144.6 (132.0, 159.0)	0.020
Weight (kg)	50.0 (42.8, 60.8)	59.1 (46.2, 71.9)	34.7 (27.0, 45.9)	< 0.001
BMI (kg/m^2^)	21.4 (20.0, 22.9)	25.6 (23.4, 28.3)	16.8 (15.2, 18.3)	< 0.001
ALT (mmol/L)	14.5 (12.0, 20.0)	24.0 (16.0, 39.0)	12.0 (10.0, 14.0)	< 0.001
AST (mmol/L)	21.0 (19.0, 24.0)	23.0 (19.0, 27.0)	22.0 (18.0,25.0)	0.002
Total cholesterol (mmol/L)	4.1 (3.5, 4.6)	4.3 (3.8, 4.9)	4.1 (3.7, 4.7)	0.072
Triglycerides (mmol/L)	0.8 (0.7, 1.2)	0.9 (0.7, 1.3)	0.8 (0.7, 1.0)	0.003
HDL cholesterol (mmol/L)	1.3 (1.1, 1.4)	1.2 (1.1, 1.4)	1.4 (1.2, 1.5)	< 0.001
LDL cholesterol (mmol/L)	2.2 (1.9, 2.5)	2.7 (2.3, 3.3)	2.1 (1.8, 2.6)	< 0.001
Free fatty acid (mmol/L)	0.4 (0.4, 0.6)	0.6 (0.4, 0.8)	0.5 (0.4, 0.6)	0.008
Glucose (mmol/L)	4.7 (4.4, 4.9)	4.9 (4.6, 5.1)	4.5 (4.3, 4.8)	< 0.001
Uric acid (mmol/L)	347.0 (294.0, 418.0)	422.0 (348.5, 515.5)	301.0 (252.5, 359.8)	< 0.001
Skin-to-liver distance (cm)	1.5 (1.2, 1.7)	1.8 (1.5, 2.1)	1.1 (1.0, 1.3)	< 0.001
ATI value (dB/cm/MHz)	0.54 (0.50, 0.62)	0.64 (0.54, 0.80)	0.51 (0.47, 0.55)	< 0.001

Data are medians with interquartile ranges reported in parentheses

*p*-values were derived from the Kruskal–Wallis *H*-test for all characteristics except for age, which was assessed using a chi-square test for comparison with the control group

*BMI* body mass index, *AST* aspartate aminotransferase, *ALT* alanine aminotransferase, *ATI* attenuation imaging

Compared with children in the control group, those with OW/OB showed significantly higher median values for height (151.0 cm vs 144.6 cm), weight (56.1 kg vs 34.7 kg), and BMI (24.3 kg/m² vs 16.8 kg/m²) (all *p* < 0.05). Within the OW/OB cohort, the OB subgroup was slightly younger than the OW subgroup (10.9 vs 11.5 years), with no significant height difference. The OB subgroup showed significantly higher median levels of LDL cholesterol (2.8 vs 2.2 mmol/L), free fatty acids (0.6 vs 0.5 mmol/L), ALT (38.2 vs 20.0 mmol/L), AST (26.6 vs 22.2 mmol/L), blood glucose (4.9 vs 4.7 mmol/L), and uric acid (435.2 vs 352.4 mmol/L). No significant differences were found in total cholesterol, triglycerides, or HDL cholesterol between the two groups (all *p* > 0.05).

### ATI value in OW/OB and control groups

**Fig. 4:**
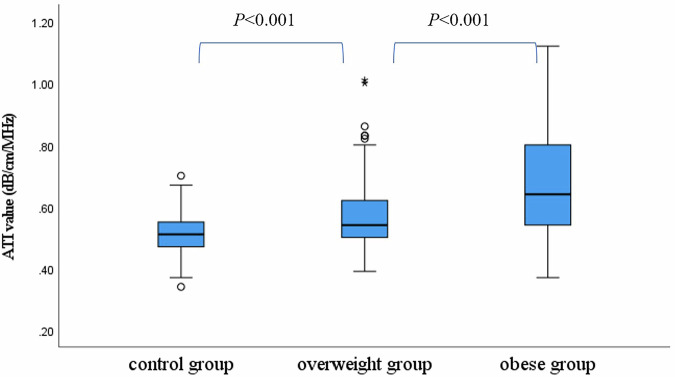
Box-and-whisker plots of the ATI values among the control, OW, and OB groups. ATI, attenuation imaging

**Fig. 5:**
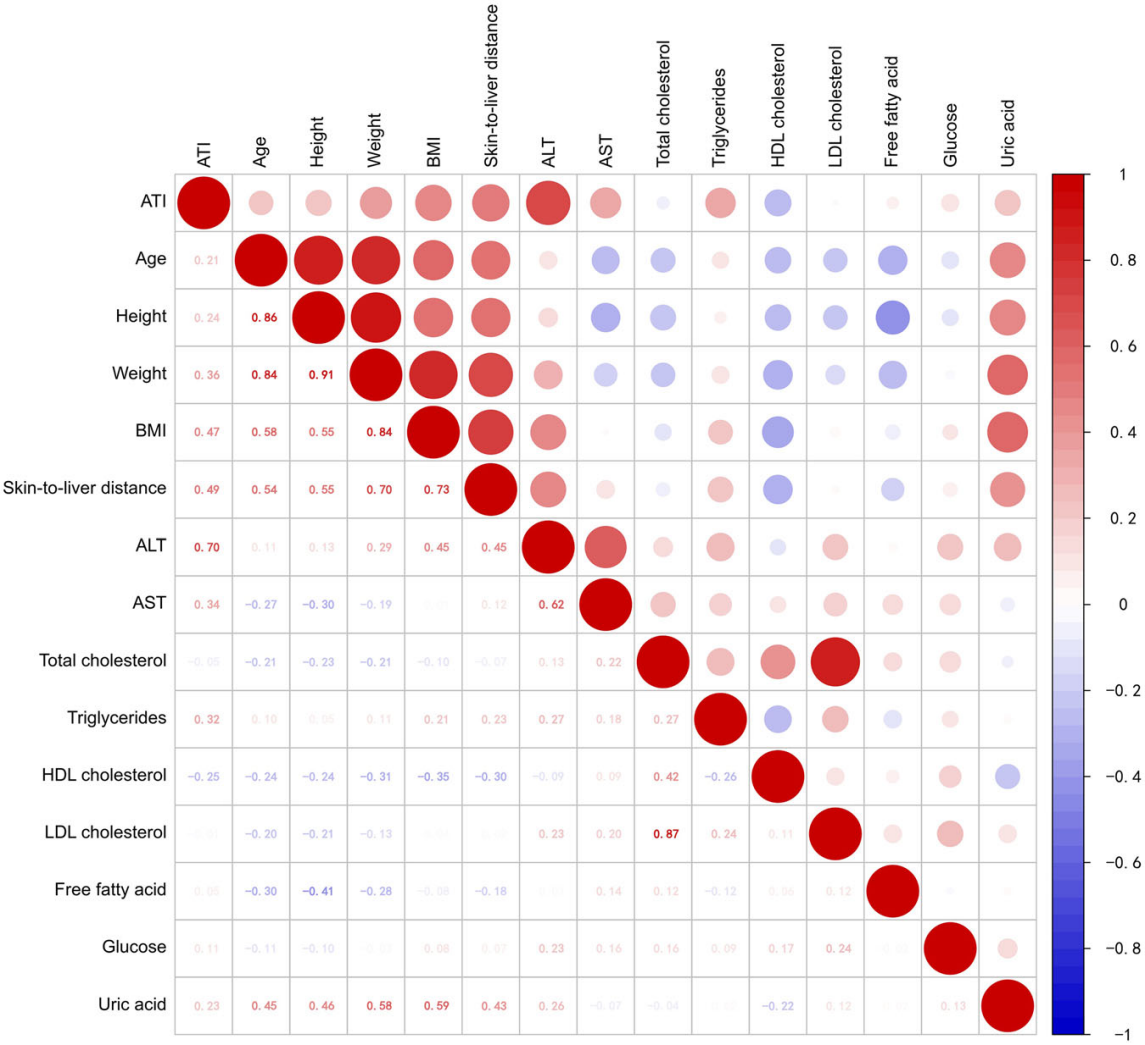
Correlation of anthropometric measures, blood biochemical profiles, US findings, and hepatic steatosis indices in OW and OB children. In this correlogram, positive correlations are expressed in red, and negative correlations are expressed in blue. The intensity of the color is proportional to the correlation coefficient. The coefficients in the expressed variables are statistically significant. ATI, attenuation imaging; BMI, body mass index; AST, aspartate aminotransferase; ALT, alanine aminotransferase

Median AC obtained with ATI for the control group, OW group, and OB group were 0.51, 0.54, and 0.64 dB/cm/MHz, respectively. Statistically significant differences were observed among all three groups (all *p* < 0.001) (Fig. 4). In the OW/OB groups, ATI values demonstrated a moderately significant positive correlation with skin-to-liver distance (*r*_s_ = 0.494, *p* < 0.001) (Fig. 5). Among anthropometric parameters, ATI values correlated weakly to moderately with age, height, weight, and BMI (*r*_s_ = 0.207, 0.237, 0.363, and 0.467; all *p* < 0.001) (Fig. 5). In contrast, no significant correlation was observed between ATI values and sex (η² = 0.031). Regarding blood biochemical parameters, ATI values showed a strong positive correlation with ALT (*r*_s_ = 0.700, *p* < 0.001), weak positive correlations with triglycerides, uric acid, and AST (*r*_s_ = 0.322, 0.234, and 0.338; all *p* < 0.001), and a weak negative correlation with HDL cholesterol (*r*_s_ = −0.252, *p* < 0.001) (Fig. 5). Of the children in the OW/OB group, 96 underwent shear wave elasticity (SWE), 98 underwent SWS, and 51 underwent SWD. Among those who completed measurements of elasticity, speed, and SWD, ATI showed no significant correlation with these parameters (*r*_s_ = 0.046, 0.060, and −0.087; all *p* > 0.05).

In the control group, 62 children also underwent SWE, 52 underwent SWS, and 34 underwent SWD measurements. The ATI values showed a moderately significant positive correlation with the SWD values (*r*_*s*_ = 0.432, *p* = 0.011), but showed no correlations with SWE and SWS (*p* > 0.05). Statistically significant correlations were observed between ATI, weight, and uric acid (all *p* < 0.05), but it is crucial to note that the correlation strengths were negligible (*r*_*s*_ = −0.126, *p* = 0.041 for weight, and *r*_*s*_ = −0.183, *p* = 0.003 for uric acid, respectively). No significant correlations were found between ATI and age, height, BMI, ALT, AST, blood glucose, or skin-to-liver distance (all *p* > 0.05).

### Correlation analysis between the ATI value and the visual grade of hepatic steatosis

**Fig. 6:**
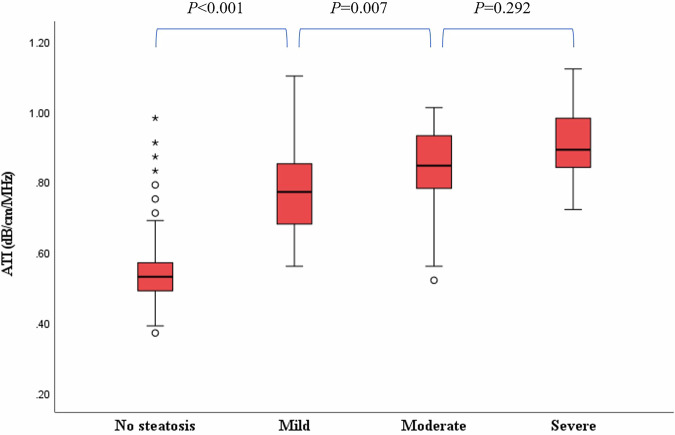
Box-and-whisker plots of the ATI values among different visual grades of hepatic steatosis. ATI, attenuation imaging

In the OW/OB groups, there are 216 children classified into Grade S0 (55.5%), 142 children classified into Grade S1 (36.5%), 22 children classified into Grade S2 (5.7%), and 9 children classified into Grade S3 (2.3%) for the visual grade of hepatic steatosis, respectively. Median ACs obtained with ATI for grades S0 (control), S1, S2, and S3 were 0.53, 0.77, 0.85, and 0.89 dB/cm/MHz, respectively, demonstrating a stepwise increase with increasing hepatic steatosis severity (Fig. 6). However, no statistically significant difference was observed between the moderate and severe subgroups. Additionally, a statistically significant positive correlation was found between the grade level of hepatic steatosis and the ATI value, with higher grades associated with greater ATI values (η² = 0.626, *p* < 0.001).

A noteworthy finding is that, despite the absence of steatosis, children in the OW/OB group exhibited a higher median ATI value than the healthy controls (0.53 vs 0.51 dB/cm/MHz). This suggests that ATI may be sensitive to grayscale US alterations associated with OB, even before the onset of overt hepatic steatosis.

## Discussion

This study investigated the distribution of ATI values in children with normal weight and those with OW/OB, and examined its correlation with the visual grading of hepatic steatosis. The results showed that ATI was significantly elevated in children with OW/OB, exhibiting a stepwise increase in line with the severity of hepatic steatosis. Furthermore, a strong positive association was confirmed between steatosis grade and ATI. By reducing inter-operator variability and providing reproducible measurements, ATI holds promise as a practical tool that could eventually complement or replace subjective US grading in clinical practice. Importantly, ATI offers distinct practical advantages that support its integration into routine clinical workflows. For children with risk factors for MASLD, incorporating ATI into routine US assessments could facilitate early identification of hepatic steatosis, prompt timely referral to specialist care, and guide the need for further diagnostic evaluation with MRI-PDFF.

The baseline ATI value in our cohort of 264 healthy children was 0.51 dB/cm/MHz, consistent with previously reported normative ranges such as those by Hwang et al (0.47–0.50 dB/cm/MHz, *n* = 40) ^15^and Song et al (0.54 dB/cm/MHz, *n* = 31) [16]. In contrast, it was notably lower than the value of 0.65 dB/cm/MHz reported by Cailloce et al (*n* = 77) [21]. Differences in baseline values compared to other studies may be attributed to several factors, including but not limited to: (1) distinct population demographics and anthropometrics; (2) the use of different US machines and transducer technologies; and (3) variations in measurement protocols and built-in software algorithms for ATI calculation. In our study, health status was defined using Chinese pediatric BMI standards, resulting in a cohort with a generally lower BMI range than that in Cailloce et al's population [21]. Further supporting this interpretation, we observed that while ATI did not correlate with age, height, weight, or BMI within the healthy control group, it exhibited weak to moderate correlations in the OW/OB group. These findings highlight the importance of adopting ethnic- and population-specific reference standards for ATI, as demographic and body composition differences may substantially influence baseline values. Therefore, multi-ethnic validation studies are essential to establish robust, generalizable normative data and to facilitate the global clinical adoption of ATI. In parallel, future efforts to establish device-specific reference ranges, or to develop standardized cross-platform calibration methods, would be important for improving the comparability and clinical application of ATI across different centers.

The correlation analysis between ATI values and visual grades of hepatic steatosis revealed several key findings. First, median ATI values showed a clear stepwise increase with higher steatosis grades, supporting ATI’s sensitivity to progressive lipid accumulation within hepatocytes and validating its role as a quantitative biomarker for grading hepatic steatosis. Second, a strong positive association was confirmed between steatosis grade and ATI (η² = 0.626, *p* < 0.001), indicating that grade level explains a substantial portion of ATI variance. However, ATI did not significantly differ between moderate and severe steatosis, suggesting a possible plateau effect or limited discriminatory power at higher levels of fat infiltration. This implies that while ATI effectively distinguishes healthy from diseased livers and mild from advanced steatosis, its precision for differentiating severe grades may be constrained. Future studies with larger cohorts in the moderate and severe categories are warranted to further investigate this phenomenon. It is noteworthy that, despite similar grayscale US appearances, the median ATI value in OW/OB children without hepatic steatosis (0.53 dB/cm/MHz) was slightly higher than that in healthy-weight controls (0.51 dB/cm/MHz). Although the absolute difference is small and its clinical significance remains uncertain, this observation suggests that ATI might offer higher sensitivity in detecting early or subtle hepatic changes that are not yet apparent on conventional US. Nevertheless, other potential contributing factors—such as abdominal wall thickness or subcutaneous fat composition—must be considered, as they could also influence the ATI measurement. Therefore, future research should involve larger-scale cohort studies with strict control of anthropometric confounders to further clarify the potential role of ATI in early MASLD screening.

The relationship between ATI values and demographic and clinical parameters appears to be context-dependent. In line with the findings of Cailloce et al [21], who reported no significant influence of age, sex, weight, or BMI on ATI, our study similarly found no significant correlation between these factors and ATI values in the control group. However, a more complex relationship was uncovered in the OW/OB group. Within this cohort, ATI values demonstrated weak to moderate positive correlations with age, height, weight, and BMI, and a moderately significant positive correlation with skin-to-liver distance. More notably, the ATI values showed a strong positive correlation with ALT and weaker positive correlations with triglycerides, uric acid, and AST. Furthermore, a weak negative correlation was observed with HDL cholesterol. This distinct correlation profile in the OW/OB group suggests that ATI is not an independent biomarker but is intricately linked to metabolic alterations and body composition, particularly in individuals with OW or OB.

This study has several limitations. First, the absence of histological verification and MRI-PDFF examinations (due to constraints in time, cost, and the need for sedation) means that our non-invasive US findings lack definitive pathological or advanced imaging correlation. Future studies should aim to include concurrent MRI-PDFF assessment alongside ATI. Establishing a strong correlation between ATI and this non-invasive quantitative MRI biomarker could provide a robust validation framework, effectively addressing the practical and ethical limitations associated with routine liver biopsy in children. Second, as a single-center investigation from South China, the findings may have limited geographic generalizability. Finally, while both US operators were highly experienced, the lack of formal intra- and inter-observer consistency tests means that potential operator-dependent variability was not quantitatively assessed. Future prospective studies should include consistency testing (e.g., ICC calculations) to further validate the reproducibility of ATI in pediatric populations.

## Conclusion

ATI values exhibit a significant stepwise increase across healthy, OW, and OB pediatric cohorts, and exhibited robust correlations with anthropometric/metabolic profiles and US steatosis severity. This makes ATI promising for screening, assessing severity, and monitoring progression in children with MASLD. However, due to the lack of validation against reference standards (MRI-PDFF or histopathology) and the population-specific nature of our cohort, broad generalization of these results is not warranted. Future multicenter studies incorporating diverse ethnic groups, standardized protocols, and direct comparisons with gold-standard modalities are essential to establish the robustness, generalizability, and ultimate clinical utility of ATI for screening, severity assessment, and longitudinal monitoring in children with MASLD.

## Abbreviations

AC: Attenuation coefficient
ATI: Attenuation imaging
BMI: Body mass index
MASLD: Metabolic-associated steatotic liver disease
MRI-PDFF: Magnetic resonance imaging of liver proton density fat fraction
OB: Obese
OW: Overweight
SWD: Shear wave dispersion
SWE: Shear wave elasticity
SWS: Shear wave speed
US: Ultrasound
